# Excellent Outcomes in Children, Adolescents, and Young Adults with Ovarian Germ Cell Tumors Treated by Either Reduced- or Standard-Dose Bleomycin

**DOI:** 10.3390/cancers15215290

**Published:** 2023-11-04

**Authors:** Meerim Park, Jin Kyung Suh, Jun Ah Lee, Hyeon Jin Park, Eun Young Park, Chong Woo Yoo, Myong Cheol Lim, Sang-Yoon Park, Byung Kiu Park

**Affiliations:** 1 Department of Pediatrics, Center for Pediatric Cancer, National Cancer Center, Goyang 10408, Republic of Korea; meerim@ncc.re.kr (M.P.); jksuh@ncc.re.kr (J.K.S.); junahlee@ncc.re.kr (J.A.L.); hjpark@ncc.re.kr (H.J.P.); 2 Biostatistics Collaboration Team, Research Core Center, National Cancer Center, Goyang 10408, Republic of Korea; 13140@ncc.re.kr; 3 Department of Pathology, Center for Gynecologic Cancer, National Cancer Center, Goyang 10408, Republic of Korea; cwy@ncc.re.kr; 4 Gynecologic Cancer Branch, Center for Gynecologic Cancer, National Cancer Center, Goyang 10408, Republic of Korea; mclim@ncc.re.kr (M.C.L.); parksang@ncc.re.kr (S.-Y.P.); 5 Department of Pediatrics, Seoul Metropolitan Seonam Hospital, Seoul 08049, Republic of Korea

**Keywords:** children, adolescents and young adults, malignant ovarian germ cell tumor, tumor marker, reduced-dose bleomycin, long-term toxicity

## Abstract

**Simple Summary:**

In a study analyzing 61 patients under the age of 39 diagnosed with malignant ovarian germ cell tumors (MOGCTs) between 2006 and 2022, the 5-year overall survival (OS) rate was 98.3%, and the event-free survival (EFS) rate was 84.9%. In total, 57 patients received bleomycin, etoposide, and cisplatin combined chemotherapy, with 44 having a reduced dose of bleomycin and 13 having the standard dose of bleomycin. The reduced bleomycin dose did not negatively impact survival. Histology and stage did not significantly impact EFS, whereas the rapid normalization of all tumor markers after surgery was associated with better EFS. Dose reduction of bleomycin did not adversely influence survival, and the overall chemotherapy-related toxicity was manageable, with no therapy-related toxic deaths.

**Abstract:**

To investigate the outcomes of children, adolescents, and young adults (AYAs) with malignant ovarian germ cell tumors (MOGCTs), we analyzed the data of 61 patients aged ≤39 years diagnosed with MOGCT between 2006 and 2022. Among 59 patients who received chemotherapy after initial diagnosis, 57 received BEP (standard dose of bleomycin with 30 units per week, *n* = 13) or bEP (reduced dose of bleomycin with 15 units/m^2^ on day 1, *n* = 44). The 5-year overall survival (OS) and event-free survival (EFS) rates were 98.3% and 84.9%, respectively. Reduced bleomycin dose did not adversely affect survival. Normalization of tumor markers within 3 months after surgery was significantly associated with better EFS (*p* < 0.01). Of the 59 surviving patients, 8 experienced surgery-related menopause, while 49 demonstrated menstrual recovery. After completion of chemotherapy, there was no significant difference in pulmonary function regarding bleomycin dose, and no overt nephrotoxicity. Approximately 60% and 25% of survivors experienced peripheral neuropathy at the end of chemotherapy and after 1 year, respectively (*p* < 0.01). Children and AYAs with MOGCT have favorable survival rates with minimal long-term toxicity, which are not influenced by a reduced bleomycin dose. Rapid normalization of tumor markers is associated with improved outcomes.

## 1. Introduction

Malignant ovarian germ cell tumors (MOGCTs) are rare, accounting for only 1–2% of ovarian malignancies, and they typically present at an early stage in children, adolescents and young adults (AYAs) [1,2]. MOGCTs comprise a heterogeneous group of histopathological variants originating from primordial germ cells in the embryonal gonad, including dysgerminoma, embryonal carcinoma, choriocarcinoma, immature teratoma (IT), yolk sac tumor (YST), and mixed MOGCT [3]. The pathogenesis of MOGCTs is not fully understood yet. Only a few studies have examined tumor development using mouse models of germ cell tumors (GCTs). Meng et al. [4] demonstrated that targeted overexpression of glial cell line-derived neurotrophic factor in undifferentiated spermatogonia promotes malignant testicular tumors using transgenic mice. Pierpont et al. [5] developed a mouse GCT model featuring germ cell-specific Kras activation and Pten inactivation. The resulting mice developed malignant, metastatic GCT composed of teratoma and embryonal carcinoma, the latter of which harboring stem cell characteristics, including OCT-4 expression. Moreover, the investigators demonstrated that genotoxic chemotherapy prolonged survival and reduced tumor size in tumor-bearing mice by eliminating the OCT4-positive cancer stem cells. Recently, it was shown that MAPK overactivation in fetal germ cells contributes to neoplastic transformation and metastatic behavior of MOGCTs using GCT mouse model carrying a germ-cell-specific BRafV600E mutation [6].

The survival rate of patients for MOGCT has significantly improved with the application of platinum-based chemotherapy, which is commonly used for testicular cancer [7,8,9]. The standard strategy for treating MOGCT involves chemotherapy with bleomycin, etoposide, and cisplatin (BEP) following primary surgery [10,11]. According to the National Comprehensive Cancer Network guidelines, the recommended dose and administration schedule of three-weekly BEP is bleomycin at 30 units per week; etoposide at 100 mg/m^2^ daily for days 1–5; and cisplatin at 20 mg/m^2^ daily for days 1–5. However, the BEP regimen is sometimes administered with a reduced dose of bleomycin and/or etoposide to mitigate serious adverse reactions [12,13]. The reduced-dose approach has been successfully established for pediatric extracranial GCTs [14]. The reduced chemotherapy consists of bleomycin at 15 units/m^2^ on day 1, etoposide at 100 mg/m^2^ daily for days 1–5, and cisplatin at 20 mg/m^2^ daily for days 1–5 (bEP). 

In this study, we investigated the treatment outcomes for MOGCT in children and AYAs, and provided updated information on treatment-related adverse events. We assessed the survival rates, recurrence rates, and treatment-related complications, including pulmonary toxicity, nephrotoxicity, and neurotoxicity. Additionally, by analyzing these outcomes, we aimed to offer insights into the optimal treatment strategies for MOGCT in the pediatric and AYA populations.

## 2. Patients and Methods

### 2.1. Patients

We analyzed the hospital records of patients, aged ≤ 39 years, who were diagnosed with MOGCT between 2006 and 2022. Patients who had received prior radiotherapy were excluded. Patients who underwent surgery and/or chemotherapy at external facilities but received adjuvant therapy in our department were included in this study.

Demographic information, tumor characteristics, cancer treatment, and long-term follow-up data were collected retrospectively. Regarding late effects, our focus was primarily on major physical health conditions, such as fertility issues, pulmonary, renal and neurological toxicities, and secondary neoplasms. This study was approved by the Institutional Review Board of the National Cancer Center (NCC2023-0097).

### 2.2. Treatment and Follow-Up

Fertility-sparing surgery was attempted whenever possible after initial diagnosis. Unilateral salpingo-oophorectomy was performed with the preservation of the contralateral ovary and of the uterus. If cystic teratoma was found in the contralateral ovary, ovarian cystectomy was undertaken with the preservation of remaining normal ovarian tissue. In cases in which fertility sparing was not feasible, radical surgery was performed. The procedure included cytologic assessment of peritoneal fluid, inspection of and biopsy of any abnormal appearing peritoneal surfaces, lymph nodes, omentum, and pelvic viscera besides bilateral salpingo-oophorectomy and hysterectomy. Patients underwent delayed surgery after neoadjuvant chemotherapy in cases in which significant surgical morbidity seemed likely or the tumors were deemed unresectable. Baseline tissue diagnosis was obtained from all patients who underwent either primary surgical resection or diagnostic biopsy. An attempt was made to measure four potential tumor markers including alpha-fetoprotein (AFP), cancer antigen 125 (CA125), CA19-9, and beta-human chorionic gonadotropin (β-hCG), but not all were tested consistently.

Following the initial investigations, all patients were risk-stratified and underwent either surgery, chemotherapy, or a combination of both. The initial chemotherapy regimen employed at the pediatric department of our center was bEP, comprising bleomycin 15 units/m^2^ on day 1, etoposide 100 mg/m^2^ daily for days 1–5, and cisplatin 20 mg/m^2^ daily for days 1–5 at 3-week intervals. In patients with renal dysfunction or obstructive uropathy, carboplatin (with an area under curve [AUC] of 7.5) was administered instead of cisplatin, while the doses of the other two agents remained unchanged. Conventional practice at our center for high-risk (HR) MOGCT was to deliver six cycles of chemotherapy after surgery, whereas four cycles were administered for intermediate-risk (IR) MOGCT. Nonetheless, the number of chemotherapy cycles varied to a limited extent, depending on the clinician’s discretion. Before 2018, patients with HR GCT received a combination of cyclophosphamide (1200 mg/m^2^ on day 1) and bEP in accordance with the AGCT01P1 trial conducted by the Children’s Oncology Group (COG). However, a subsequent COG report did not establish a beneficial role in terms of survival with the addition of cyclophosphamide [15]. Moreover, cyclophosphamide is known to be gonadotoxic and potentially carcinogenic. Therefore, since 2018, cyclophosphamide had no longer been included in the treatment plan for patients with HR GCT.

Our treatment strategy typically involves a reduced bleomycin regimen, as described above. However, our cohort included 13 patients who received a standard dose of bleomycin (BEP or CyBEP), including those who had initially been treated in another hospital and transferred to our institution, and those who had been treated at another department in our institution, but were later followed up in the pediatric department.

For patients who agreed, GnRH agonists (GnRHa) were co-administered during chemotherapy to those who underwent fertility-sparing surgery, which was defined as surgery in which the uterus and at least part of one ovary were left in situ.

After completing chemotherapy, we performed regular check-ups that included clinical examinations, measurements of blood tumor markers, and imaging studies every 3 months in the first year. Subsequently, we gradually increased the interval between check-ups. During the follow up, we monitored patients’ menstruation status and assessed their pulmonary and renal functions. Adverse events were defined using the modified Common Terminology Criteria for Adverse Events (CTCAE) version 4.0.

### 2.3. Statistical Analysis

Baseline characteristics are summarized as the mean ± standard deviation or median (range) for continuous variables, and frequency (%) for categorical variable. Patients with an increase in tumor size, new lesions, and/or rising tumor markers are classified as having progressive disease (PD). Relapse is defined as the reappearance of a disease after it has disappeared. Event-free survival (EFS) was defined as the interval from the time of diagnosis to PD/relapse, occurrence of second malignancy, or death from any cause, whichever occurred first, and overall survival (OS) was defined as the interval from the time of diagnosis to death from any cause. Growing teratoma syndrome (GTS) was not considered as an event. The 5-year survival rate was estimated using the Kaplan–Meier method, and the survival rates between the groups were compared using the log-rank test. The correlation between the proportion of the YST component in a tumor specimen and serum AFP levels was analyzed using Spearman’s correlation method. The Contal and O’Quigley’s methods were used to explore the cut-off value for tumor marker normalization time after surgery to achieve a superior EFS, and the association between EFS and clinical variables was analyzed using the Cox proportional hazards model. Long-term chemotherapy-related toxicities such as lung function, renal function, and neurotoxicity were summarized and compared between the groups. Toxicity at paired time points was compared using the Wilcoxon signed rank and McNemar’s tests. A Wilcoxon rank sum test was performed to compare toxicity according to the dose of bleomycin and number of chemotherapy cycles. All statistical analyses were performed using SAS version 9.4 (SAS Institute Inc., Cary, NC, USA) and R version 4.1.1 (R Foundation for Statistical Computing, Vienna, Austria).

## 3. Results

### 3.1. Clinical Characteristics

This study included 61 patients diagnosed with MOGCT between 2006 and 2022. The patients had a median age of 20.9 (range: 5.3–39.2) years. Among these, seven were premenarchal. Table 1 summarizes the patient characteristics. The median duration of follow-up was 60.8 (range: 1.6–205.5) months from the time of diagnosis. One patient with mixed GCT had androgen insensitivity syndrome.

Patients were staged as follows: 32 were categorized into stage I (52.5%), 22 into stage III (36.1%), and 7 into stage IV (11.5%). The most common histologic type was mixed GCT (*n* = 23, median age of 17.0 years), followed by IT (*n* = 16, median age of 21.5 years), YST (*n* = 11, median age of 23.3 years), and dysgerminoma (*n* = 11, median age of 23.0 years). In total, 54 patients were examined for tumor markers at the time of diagnosis, although the tested markers were not uniform among the patients. Among 47 patients checked for CA-125, 43 (91.5%) showed an elevated CA-125 with a median value of 232 U/mL. Serum AFP was measured in 44 patients, and in 29 (65.9%) it was beyond the normal range. Among patients with elevated AFP levels, the median was highest in patients with YST (42,518 U/mL), followed by those with mixed GCT (14,761 U/mL), and dysgerminoma (1850 U/mL). Thirty-four patients were examined for CA19-9 and seven (20.6%) (four IT + three mixed GCT) had values greater than the normal range. In mixed GCT, the proportion of YST components in a tumor specimen demonstrated positive correlation with serum AFP levels (*r* = 0.575). 

### 3.2. Initial Therapeutic Strategy 

All patients underwent surgical resection of the tumor. Three patients (4.9%) (two stage IV and one stage IIIC) initially underwent biopsy, and then underwent definitive surgery after receiving two or three cycles of neoadjuvant chemotherapy. 

Chemotherapy was administered to all patients after the initial diagnosis except in two: one with grade 1, stage IC IT and the other with stage IA dysgerminoma who did not receive chemotherapy. The former survived without recurrence for over 4 years, whereas the latter experienced recurrence with multiple pelvic masses after 6 months of radical surgery (patient No. 3 in Table 2). Among the 59 patients who received chemotherapy after the initial diagnosis, 57 (96.6%) received BEP or bEP with or without cyclophosphamide, with bEP in 26 (44.1%), CybEP in 18 (30.5%), BEP in 11 (18.0%), and CyBEP in 2 (3.3%), respectively. Two other patients (3.3%) received paclitaxel and carboplatin, and EMA-CO (etoposide, methotrexate, actinomycin D, cyclophosphamide, vincristine), for each. After surgery, chemotherapy was initiated within a median of 16 days (range: 10–35 days) and the median number of chemotherapy cycles administered was four (range: 1–8). Tumor markers were normalized after a median of three cycles (range: 0–5 cycles) of chemotherapy. Approximately 84% of the patients exhibited a normal tumor marker upon completing three cycles of chemotherapy.

After receiving initial chemotherapy, seven patients (11.5%) underwent a second-look surgery because of the possible residual viable tumors, as indicated by computer tomography or magnetic resonance imaging findings. Of these, four patients exhibited viable malignant GCT, two exhibited necrotic tissues only, and one exhibited a mature teratoma.

### 3.3. Outcomes and Prognostic Variables

**Figure 1:**
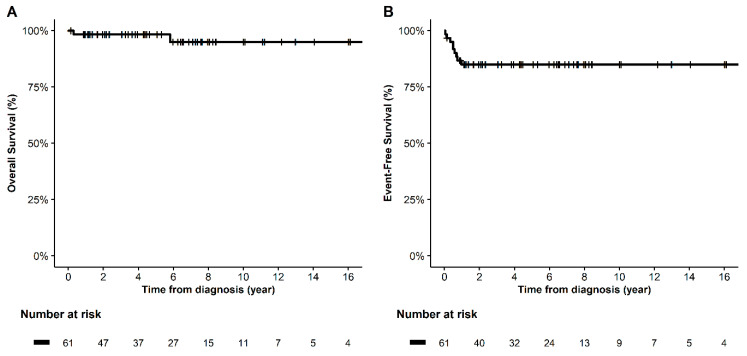
Kaplan–Meier survival curves of (**A**) overall survival and (**B**) event-free survival in all patients.

The estimated 5-year OS and EFS rates were 98.3% and 84.9%, respectively (Figure 1). Relapse or PD after initial chemotherapy occurred in 9 of 61 patients (Table 2). Overall, two patients succumbed to death due to PD: one (patient No. 2 in Table 2) displayed poor responsiveness to chemotherapy from the beginning, and tumor cells obtained during upfront surgery carried PIK3CA and TP53 mutations and were strongly positive for programmed death-ligand 1 (PD-L1). These molecular changes might have contributed to the intrinsic chemo-resistance. Another patient (patient No. 5 in Table 2) with stage IV mixed GCT had been previously diagnosed with androgen insensitivity syndrome. Despite chemotherapy and surgery, the teratoma component continued to recur. Final tumor recurrence occurred in the psoas muscle, and pathologic exam revealed it to be leiomyosarcoma. The patient underwent subtotal resection and radiation therapy, but eventually died due to disease progression. Genomic analysis of the tumor was not conducted.

**Figure 2:**
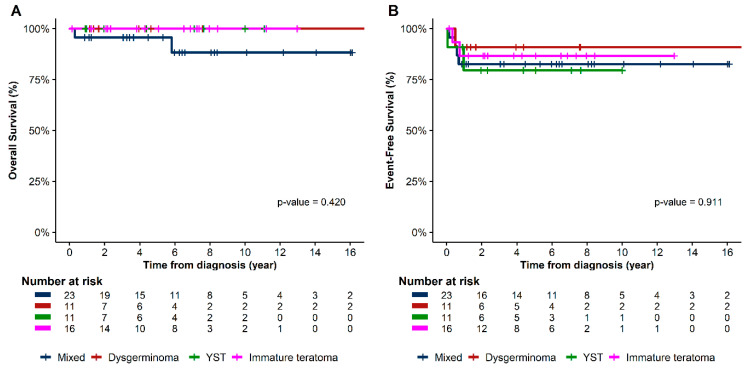
Kaplan–Meier survival curves of (**A**) overall survival and (**B**) event-free survival based on histology.

**Figure 3:**
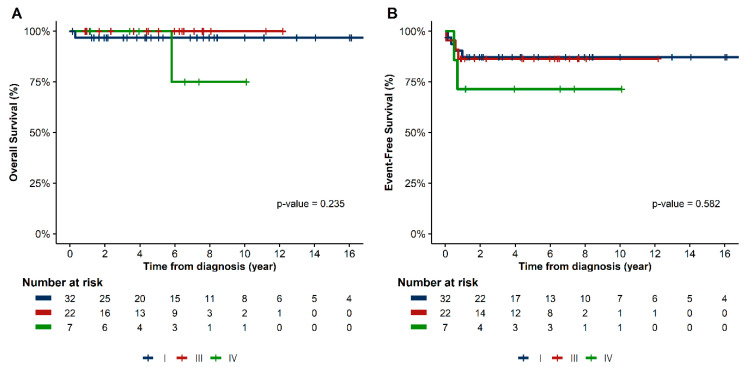
Kaplan–Meier survival curves of (**A**) overall survival and (**B**) event-free survival based on FIGO stage. FIGO, Fédération Internationale de Gynécologie et d’Obstétrique.

**Figure 4:**
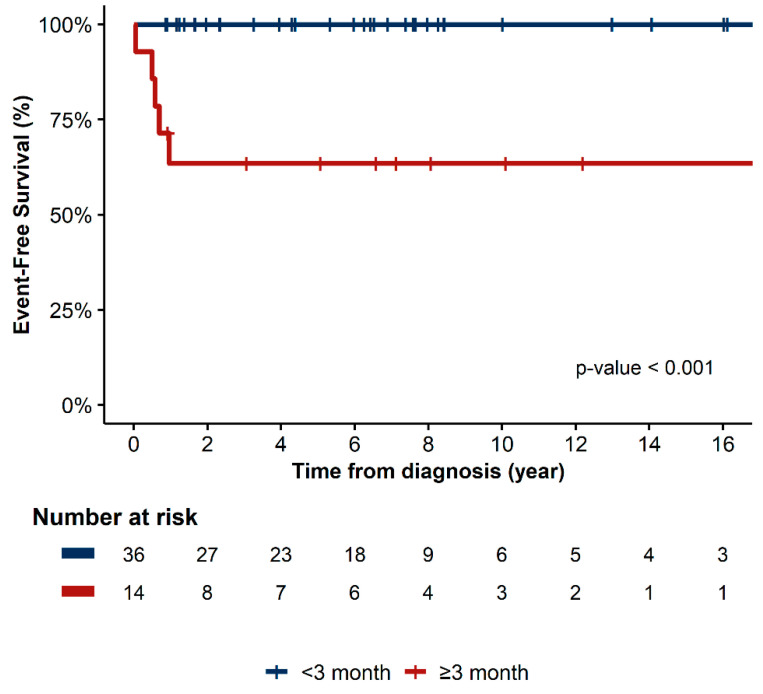
Event-free survival based on the time between surgery and tumor marker normalization.

Chemotherapy was well tolerated, and no toxic death occurred. Based on the histologic subtype, the patients’ estimated 5-year EFS were as follows: dysgerminoma, 90.9%; IT, 86.7%; mixed GCT, 82.6%; and YST, 79.5% (*p* = 0.91) (Figure 2). In terms of stage, the EFS for patients with stages I, III, and IV were 87.2%, 86.4%, and 71.4%, respectively (*p* = 0.58) (Figure 3). In the univariate analysis, age, histology, stage, bleomycin dose, addition of cyclophosphamide to bEP/BEP, and number of chemotherapy cycles were not associated with EFS. Only the rapid normalization of all tumor markers after surgery was significantly associated with EFS. Patients who experienced normalization of tumor markers within 3 months after surgery had a higher EFS than that of those for whom this normalization occurred did after 3 months (*p* < 0.01) (Figure 4).

GTS was detected in nine patients (six mixed GCT and three IT) at a median of 7.7 months after diagnosis, with four developing GTS during chemotherapy. Growing teratomas were surgically removed in all patients, and pathologic exam revealed mature teratoma. No further recurrence of GTS was detected in these patients. Gliomatosis peritonei were diagnosed in two patients with IT; one patient with grade 2 IT developed it during chemotherapy, whereas the other patient with grade 3 IT developed it 6 months after completing chemotherapy. The former experienced recurring episodes of gliomatosis peritonei.

### 3.4. Reproductive Outcomes and Long-Term Chemotherapy-Related Toxicity

Among the 59 surviving patients, menopause was an obvious consequence of radical surgery in 8 (13.6%). Among the remaining 51 patients, only 2 did not experience menstrual recovery. Among them, one patient with dysgerminoma underwent left salpingo-oophorectomy, and did not receive GnRHa during chemotherapy. The patient is currently 10 years old and completed cancer treatment a year ago. Thus, she still has a chance of menarche. Another patient with mixed GCT underwent right oophorectomy with pelvic and paraaortic lymph node dissection. She completed six cycles of CybEP chemotherapy with concomitant GnRHa 6 years ago and is currently 34 years old.

Of the 58 survivors (excluding 1 patient who did not receive chemotherapy), 42 patients received GnRHa, and anti-Mullerian hormone (AMH) levels were mostly measured in these patients. The medians of AMH level at the completion of chemotherapy and 1 year after treatment were 0.03 ng/mL and 1.55 ng/mL, respectively. At a median of 45 months after treatment, the median AMH level was 2.25 ng/mL. The mean period from the end of treatment to menstrual resumption was 7.07 months in patients who received GnRHa. However, the data were insufficient to calculate the mean values for patients who did not receive GnRHa.

Long-term toxicities were evaluated in the patients who received first-line chemotherapy only. Long-term chemotherapy-related toxicities were minimal, with few observed complications. No secondary neoplasms have been reported. Pulmonary function test data were available for 39 patients. The means of forced vital capacity (FVC), forced expiratory volume in one second (FEV1), FEV1/FVC, and diffusion capacity of the lung for carbon monoxide (DLCO) at the end of therapy were 88.0% ± 14.6%, 90.2% ± 15.2%, 98.0% ± 11.0%, and 58.5% ± 14.0%, respectively. At 1 year after completion, the means of FVC, FEV1, FEV1/FVC, and DLCO were 84.3% ± 11.0%, 83.7% ± 12.5%, 102.0% ± 11.0%, and 77.1% ± 13.2%, respectively. DLCO, which were below normal at the end of treatment and significantly increased to a near-normal level after 1 year of treatment (*p* = 0.02). A total of 11 of the 38 tested (29%) patients showed a restrictive pattern at the end of chemotherapy, and 7 of the 15 tested (47%) at 1 year after completion of chemotherapy (*p* = 0.32). None of the patients exhibited an obstructive pattern. The PFT profiles of patients who received reduced doses of bleomycin (bEP and CybEP) were not significantly different from those of patients who received standard doses of bleomycin (BEP and CyBEP) (Table 3). After completing chemotherapy, a significant difference in FVC and FEV1 was observed between cycles ≤ 4 and >4 (*p* < 0.01, both); however, the median of FVC and FEV1 in both groups were within the normal range (Table 3). Nonetheless, 1 year after chemotherapy completion, the difference in FVC and FEV1 between these two groups was no longer significant (*p* = 1.0 and 0.6, respectively). Patients who underwent chemotherapy cycles of >4 had a greater likelihood of experiencing restrictive pattern of PFT abnormalities than those who received chemotherapy cycles of ≤4 (*p* = 0.07). However, after 1 year of completing chemotherapy completion, the difference was no longer significant (*p* = 1.0). Interstitial pneumonitis or pulmonary fibrosis were not observed.

The renal function was evaluated in 47 patients by measuring the estimated glomerular filtration rate (eGFR). The mean eGFR was 124.5 ± 30.0 mL/min/1.73 m^2^ at the time of chemotherapy completion, and 122.6 ± 20.4 mL/min/1.73 m^2^ at 1 year after completion. With respect to the median eGFR, values in the reduced-dose and standard-dose bleomycin groups were not different both at the completion of therapy and 1 year after therapy, and they were all within the normal ranges (Table 4). Similarly, values in the ≤4 cycle group and >4 cycle groups were not different at both time points and were also within the normal ranges. No chemotherapy-related renal adverse events of CTCAE grade II or higher were reported upon the completion of chemotherapy. None of the patients reported having developed chronic kidney disease.

Before or at the completion of chemotherapy, 59.6% of the patients reported experiencing numbness and/or a tingling sensation in their hands and/or feet, which corresponded to CTCAE Grade II. However, there were no cases of CTCAE Grade III or IV peripheral neurotoxicity. At 1 year after completion of chemotherapy, the proportion of patients with peripheral neuropathy decreased significantly to approximately 25% (*p* < 0.01). The patients subject to peripheral neuropathy exhibited gradual improvements over time. No significant difference in the incidence of peripheral neuropathy was observed between the ≤4 cycle and >4 cycle groups at the end of chemotherapy completion and 1 year after completion.

## 4. Discussion

This study included 61 patients with MOGCT with a median age of 20.9 years. Most patients were categorized as stage I (52.5%), followed by stage III (36.1%) and stage IV (11.5%). Mixed GCT was the predominant histologic type. All patients underwent surgical management, and most received chemotherapy soon after tumor resection. Approximately 97% of the patients received BEP or bEP with or without cyclophosphamide. The median number of chemotherapy cycles administered was four, and tumor marker normalization was achieved within a median of three cycles.

This study confirmed the excellent prognosis of MOGCT, with the estimated 5-year OS and EFS rates of 98.3% and 84.9%, respectively. Among the 61 patients, 7 experienced relapse and 2 experienced PD. Eventually, all seven recurrent cases were successfully rescued, while two patients with PD died, indicating that the outcomes of recurrent disease were much more favorable than those of PD in our cohort. MOGCTs can be cured, a small percentage experience relapse and require salvage therapy. This may include proper salvage surgery alone with either standard- or high-dose salvage chemotherapy. The most commonly used standard-dose salvage regimen includes a combination of cisplatin plus ifosfamide with paclitaxel (TIP) or vinblastine (VelP) [16]. Patients resistant to cisplatin-based treatment can also be treated with vincristine/actinomycin D/cyclophosphamide, paclitaxel/gemcitabine, or topoisomerase I-based chemotherapy as salvage therapy [17,18]. Targeted treatments/immunotherapy for GCTs have been reported using EGFR family, PI3K/AKT/mTOR, c-KIT, multiple tyrosine-kinase inhibitors, and antiangiogenic agents [19]. Ongoing or recently completed clinical trials with targeted therapies or immunomodulators for GCTs include brentuximab (NCT01851200), pazopanib (NCT01743482), atezolizumab (NCT02458638), olaparib (NCT02533765), and sirolimus + erlotinib (NCT01962896). Recently, sempervirine, an RNA polymerase I inhibitor, have demonstrated anti-cancer effects in GCTs [20]. However, thus far, no targeted treatment for GCTs has proved to be effective in clinical use. The reasons for this may be due to the diversity of the subtypes of GCTs or the difficulty in recruiting a sufficient number of patients for clinical trials due to the low rate of refractory GCTs.

In our series, fertility-sparing surgery was attempted whenever possible after initial diagnosis and 83.6% of patients achieved complete surgical resection. Some studies have shown that maximal debulking surgery is significantly associated with improved overall survival [21,22], while others have not [23,24]. Considering the chemotherapy-sensitive nature of MOGCTs, fertility-sparing cytoreductive surgery seems reasonable for patients in advanced stage. Currently, there is a trend towards less extensive surgical procedures for young women over time was observed, without negatively impacting cancer-specific survival [25]. For patients with extensive disease, neoadjuvant chemotherapy followed by delayed surgery may be a reasonable option when initial debulking is not feasible or precludes fertility-sparing surgery [26]. This is currently not the standard of care, but it deserves future study.

Histology and stage did not significantly affect EFS, but rapid normalization of all tumor markers was associated with better EFS. Patients who achieved normalization of tumor markers within 3 months after surgery exhibited higher EFS rates than those who did so after 3 months (*p* < 0.01). In adults with HR testicular and extragonadal GCT, AFP decline was identified as an important prognostic factor, justifying treatment intensification in cases of insufficient AFP decline after one chemotherapy course [27,28]. Studies on tumor marker decline in pediatric patients are quite limited. Fresneau et al. [29] investigated AFP decline in 179 patients with non-seminomatous GCT, aged ≤ 18 years, and they reported that the risk of progression or relapse is marginally increased with a small AFP decline during the first 21 days. More recently, the Malignant Germ Cell International Consortium reported data on 131 patients with GCT under 21 years of age suggesting that a satisfactory AFP decline after therapy is significantly associated with the cumulative incidence of relapse, but not with OS [30]. The authors presumed that the lack of association between AFP decline and OS is attributed to successful salvage therapy and a small number of events. The findings of previous studies, as well as the present study, indicate that tumor marker decline is a valuable indicator for identifying patients at high risk of relapse and offering altered therapy early before relapse.

In this study, approximately 11% of the patients underwent a second-look surgery after chemotherapy, revealing four cases with viable malignant GCT, two with residual necrotic tissues, and one with mature teratoma. It is important to note that residual teratomas carry the risk of malignancy. To mitigate this risk, debulking surgery is recommended, which not only allows histologic diagnosis, but also helps determine the suitable treatment for the specific transformation. GTS is well known in male patients with GCTs, whereas ovarian GTS is less commonly reported [31,32]. Bentivegna et al. [33] reported the largest series of GTS in MOGCT. In their study, 19% of patients with IT experienced GTS. The mean delay between IT and GTS was 7 months. In our study, GTS was detected in nine patients: five with IT and four with mixed GCT containing IT, occurring at a median 7.7 months after diagnosis, and four patients d eveloped GTS during chemotherapy. Growing teratomas were surgically removed in all patients, with pathology revealing mature teratoma. No further recurrence of GTS was detected in these patients. If the patients’ original tumors had a teratoma component and they subsequently had residual or recurrent growing masses but their tumor markers remained normal, it may not necessarily indicate disease progression. Chemotherapeutic retroconversion and growing teratomas are not so rare. Awareness of growing teratomas is essential to avoiding unnecessary chemotherapy [34]. Lesions can remain stable for years. However, once they grow, histologic confirmation through surgical resection is necessary to rule out relapse or malignant transformation. Although rare, sarcomas, carcinoids, adenocarcinomas, and primitive neuroectodermal tumors have been previously described as malignant transformation patterns in GTS [35,36]. Gliomatosis peritonei, the peritoneal and omental implantation of mature glial tissues, is an extremely rare entity that was diagnosed in two of our patients with IT. Overall, 7 of 16 patients with IT (43.8%) experienced GTS or GP, indicating that the occurrence of GTS or GP in patients with IT should always be taken into consideration, with regular patient follow-up being a necessity.

Some trials on GCT showed worse results after reducing the doses of bleomycin and/or etoposide [37,38]. However, dose reduction of bleomycin did not influence survival in our study, which needs to be confirmed in prospective studies using a larger and homogeneous population. The BEP regimen remains the gold standard for treatment of adult and postpubertal pediatric patients with GCT [10,11]. A group of pediatricians suggested a chemotherapy regimen using bEP to reduce the risk of long-term side effects (especially pulmonary sequelae), while ensuring high survival rates [14]. In our study, among the 57 patients who received an EP-based regimen with bleomycin, 44 (77.2%) received reduced-dose bleomycin. Our findings demonstrated that reduced dose bleomycin for children and AYAs with MOGCTs did not adversely affect survival, while also alleviating concerns about side effects associated with standard dose bleomycin.

No therapy-related death was reported, and long-term chemotherapy-related toxicity was minimal, with no case of secondary neoplasm. At the end of treatment, the FVC, FEV1, and FEV1/FVC were well preserved, while the DLCO was reduced to 58.5% ± 14.0%. However, 1 year after treatment completion, there was a noticeable improvement in DLCO, with a rise to 77.1% ± 13.2%. Pulmonary function abnormalities, particularly reduced DLCO and, in more severe cases, a restrictive ventilation defect causing arterial hypoxemia, are the most common concerns [39]. In agreement with these findings, our study revealed a decline in DLCO, which subsequently recovered to near-normal value in 1 year. PFT results were then separately analyzed according to bleomycin dose and number of chemotherapy cycles. The PFT profiles of the patients who received a reduced dose of bleomycin did not differ from those of the patients who received a standard dose of bleomycin (Table 3). Among the FVC, FEV1, FEV1/FVC, and DLCO values examined, only DLCO was below normal at the end of therapy in both the reduced- and standard-dose bleomycin groups. However, DLCO increased to a near-normal value in 1 year. Similar to PFT results stratified by bleomycin dose, both the ≤4 cycle and >4 cycle groups demonstrated normal FVC, FEV1, and FEV1/FVC values and decreased DLCO at the end of therapy. DLCO subsequently returned to near-normal value after 1 year of therapy completion. No significant difference in four PFT parameters were observed between the groups, except for FVC and FEV1 values at the end of therapy.

Overt nephrotoxicity was not observed in this study, although approximately 60% of the patients encountered peripheral neuropathy that eventually improved with time. Surgery-related menopause occurred in 13.6% of the patients. Excluding these patients, approximately 96% of patients resumed regular menstruation regardless of GnRHa use. The percentage of menstrual recovery after chemotherapy among patients with MOGCT has been reported to be 79–99% [40]. In a previous study, fertility-sparing surgery combined with BEP chemotherapy demonstrated a success rate of 75% in achieving pregnancies [41]. Patients with MOGCT generally experience relatively low rates of premature ovarian insufficiency after chemotherapy owing to the modest gonadotoxicity of the BEP regimen and their young age, which ensures a robust ovarian reserve. However, few patients experiencing early menopause at a young age may encounter fertility challenges. To preserve fertility and minimize the risk of early ovarian insufficiency, the co-administration of GnRHa during chemotherapy is an additional option alongside fertility-sparing surgery [42]. With respect to the occurrence of ovarian insufficiency, a cross-analysis conducted by Choi et al. [43] showed a significant difference between the GnRHa and non-GnRHa groups. In our series, patients who received GnRHa co-administration exhibited a gradual increase in serum AMH levels, from 0.03 ng/mL at the completion of chemotherapy and 1.55 ng/mL at 1 year after treatment to 2.25 ng/mL at a median of 45 months after treatment. Randomized trials are necessary to verify the potential advantages of utilizing GnRHa to preserve ovarian reservoirs in children and AYAs with MOGCT.

The management of stage I disease remains a subject of debate [44,45]. For stage I malignant testicular GCT, surgery alone results in a cure rate of 75%, and salvage chemotherapy is successful even in cases of recurrence [46]. Similarly, COG recently reported that 50% of patients with stage I ovarian GCT who were treated by surgery alone experienced recurrences, but salvage therapy led to a 4-year OS rate of 96% [47]. In contrast, a meta-analysis supported the use of adjuvant chemotherapy in stage I ovarian IT, as it has been shown to decrease mortality rates compared to surveillance [45]. In our study, of the two patients with stage I GCT who did not receive chemotherapy, one (patient No. 3 in Table 2) relapsed with multiple pelvic masses. It is premature to make a decision based on hitherto knowledge on the optimal therapy for stage I MOGCT. 

This study had limitations owing to its small sample size and retrospective design. This may have contributed to non-significant findings when analyzing multiple factors, including the effect of reducing the dose of bleomycin on event-free survival (EFS). To verify our results, further prospective studies using a larger and more homogeneous population are needed. Moreover, PFT was not performed according to a predetermined format, thus hindering the verification of beneficial effects of reduced bleomycin dose on pulmonary function. Nonetheless, it is the largest single-center study investigating the outcomes for MOGCT in children and AYAs, with a relatively long follow-up period.

## 5. Conclusions

Our study highlights the excellent prognosis of MOGCT in children and AYAs. Histology and stage did not have a significant impact on EFS, whereas the rapid normalization of all tumor markers after surgery was associated with better EFS. Dose reduction of bleomycin did not adversely influence survival, and the overall chemotherapy-related toxicity was manageable, with no therapy-related toxic deaths. We suggest that bEP regimen may be employed even in AYAs with MOGCT. Further studies are warranted to verify our findings through randomized trials and to investigate the implications of serum marker changes in determining the chemotherapy strategies and predicting the ultimate outcomes.

## Figures and Tables

**Table 1:** Patient characteristics.

	*n* = 61
Median age at diagnosis (years)	20.9 (5–39)
Age < 10	3 (4.9%)
Age 10–19	25 (41.0%)
Age 20–29	22 (36.1%)
Age 30–39	11 (18.0%)
Histology	
Dysgerminoma	11 (18.0%)
Immature teratoma	16 (26.2%)
Yolk sac tumor	11 (18.0%)
Mixed	23 (37.7%)
FIGO stage	
I	32 (52.5%)
II	0 (0%)
III	22 (36.1%)
IV	7 (11.5%)
β-hCG	(*n* = 38)
Elevated	14 (36.8%)
Median (range), mIU/mL	116.6 (3.1–16,000)
Not elevated	24 (63.2%)
AFP	(*n* = 44)
Elevated	29 (65.9%)
Median (range), U/mL	15,374 (16.6–360,186)
Not elevated	15 (34.1%)
CA125	(*n* = 47)
Elevated	43 (91.5%)
Median (range), U/mL	232 (39.3–1924)
Not elevated	4 (8.5%)
CA19-9	(*n* = 34)
Elevated	7 (20.6%)
Median (range), U/mL	90 (56.8–664)
Not elevated	27 (79.4%)
Surgery	
Upfront surgery only	51 (83.6%)
Biopsy + delayed surgery	3 (4.9%)
Upfront surgery + salvage surgery	7 (11.5%)
First-line chemotherapy	
bEP	26 (42.6%)
BEP	11 (18.0%)
CybEP	18 (29.5%)
CyBEP	2 (3.3%)
Others	2 (3.3%)
No chemotherapy	2 (3.3%)

AFP, alpha fetoprotein; BEP, bleomycin 30 units per week + etoposide + cisplatin; bEP, bleomycin 15 units/m^2^ on day 1 + etoposide + cisplatin; b-hCG, beta-human chorionic gonadotropin; CA-125, cancer antigen 125, CA-19-9, cancer antigen 19-9; CybEP, cyclophosphamide + bleomycin 15 units/m^2^ on day 1 + etoposide + cisplatin, CyBEP, cyclophosphamide + bleomycin 30 units per week + etoposide + cisplatin; FIGO, Fédération Internationale de Gynécologie et d’Obstétrique.

**Table 2:** Characteristics of patients with progressive or recurrent disease (*n* = 9).

No.	Age at Diagnosis (Years)	Histology	FIGO Stage	Surgery	Adjuvant Chemo	Time from Diagnosis to Progression or Recurrence (Months)	Site of Recurrence	Treatment after Progression or Recurrence	Follow-Up Period (Months)	Status at Last Follow-Up
1	15	Mixed (YST 90% + embryonal ca 10%)	IIIB	RSO, LOC, omentectomy	BEP (4)	6 (R)	Mass anterior to LO	CybEP (2) → TIC (4)	52+	NED
2	25	Mixed (dysgerminoma 60% + embryonal ca 40%)	IA	LSO	bEP (2)	1 (PD)	Peritoneal LN	TIP (1) → GDC (1)	3	DOD
3	34	Dysgerminoma	IA	TLH, BSO, PLND, PALND, appendectomy	No chemo	5 (R)	Pelvic mass	BEP (1) → surgery → bEP (2) →relapse → bEP (2) → TIC (6)	52+	NED
4	22	YST	IC	RSO	TC (3)	11 (R)	Peritoneal seeding, liver	CybEP (2) → surgery→ CybEP (4)	119+	NED
5	30	Mixed (IT + YST + embryonal ca + dysgerminoma) with androgen insensitivity syndrome (SRYgene mutation+)	IV	Bilateral gonadectomy, PLND, PALND	BEP (3), RT	6 (PD)	Pelvic mass	TIC (2) → Gemcitabine + oxaliplatin (1) → laparotomy →relapse (liver) → tumorectomy → relapse (neck lymph node) → excision →relapse (psoas muscle) → subtotal resection →radiation therapy	69	DOD
6	17	Mixed (YST 95% + mature teratoma 5%)	IV	TAH, BA, PLND, PANLD, omentectomy	BEP (4)	8 (R)	Peritoneal seeding	BEP (2) → EP(3) →TIC (6) → relapse → surgery → TIC (1) →ICE (2) → HDCT/autoSCR → relapse → surgery GOP (8) → oral VP-16	37+	NED
7	29	IT (grade 2)	IC	RSO	-	4 (R)	Peritoneal seeding, inguinal LNs	Surgery → bEP (6)	83+	NED
8	14	IT (grade 2) with AFP elevation	unknown	LSO, omentectomy	BEP (6)	9 (R)	Peritoneal seeding	Surgery → ICE (1) → TIP (2) → surgery, HIPEC → pathology: gliomatosis + immature teratoma → IFN-2a	131+	NED
9	26	YST	IIIC	RSO	-	1 (R)	Peritoneal seeding	bEP (3) → surgery → bEP (3)	8+	NED

AFP, alpha fetoprotein; BA, bilateral adnexectomy; BEP, bleomycin (standard dose) + etoposide + cisplatin; bEP, bleomycin (reduced dose) + etoposide + cisplatin; BSO, bilateral salpingo-oophorectomy; DOD, died of disease; EP, etoposide + cisplatin; FIGO; Fédération Internationale de Gynécologie et d’Obstétrique; GDC, Gemcitabine + docetaxel + carboplatin; GOP, gemcitabine + oxaliplatin + cisplatin; HDCT/autoSCR, high dose chemotherapy and autologous stem cell rescue; HIPEC, hyperthermic intraperitoneal chemotherapy with cisplatin; ICE, ifosfamide + carboplatin + etoposide; IFN-2α, interferon-2 alpha; IT, immature teratoma; LN, lymph node; LO, left ovary; LSO, left salpingo-oophorectomy; NED, no evidence of disease; PALND, paraaortic lymph node dissection; PD, progressive disease; PLND, pelvic lymph node dissection; R, relapse; RSO, right salpingo-oophorectomy; TAH, total abdominal hysterectomy; TC, taxol + carboplatin; TIC, taxol + ifosfamide + carboplatin; TIP, taxol + ifosfamide + cisplatin; TLH, total laparoscopic hysterectomy; YST, yolk sac tumor.

**Table 3:** Comparison of pulmonary function profiles at the completion of chemotherapy and 1 year post-chemotherapy.

		Reduced Bleomycin (*n* = 32)	Standard Bleomycin (*n* = 7)	*p*-Value
At the completion of chemotherapy	FVC	88 (67–121)	81 (55–105)	0.23
	FEV1	89.5 (66–126)	88 (53–112)	0.56
	FEV1/FVC	1 (0.8–1.3)	0.9 (0.9–1)	0.21
	DLCO	61 (1–78)	68 (46–81)	0.20
One year post-chemotherapy	FVC	83 (72–105)	75.5 (74–77)	0.30
	FEV1	84 (63–102)	83.5 (81–86)	0.93
	FEV1/FVC	1 (0.8–1.2)	0.9 (0.9–1)	0.14
	DLCO	76 (50–99)	80 (80–80)	0.66
		**Cycles of chemotherapy ≤ 4** **(*n* = 17)**	**Cycles of chemotherapy > 4** **(*n* = 22)**	***p*-value**
At the completion of chemotherapy	FVC	97 (55–121)	84 (63–103)	<0.01 *
	FEV1	100.5 (53–126)	84.5 (66–104)	<0.01 *
	FEV1/FVC	1 (0.9–1.1)	1 (0.8–1.3)	0.67
	DLCO	62.5 (1–81)	61 (43–77)	0.39
One year post-chemotherapy	FVC	83 (72–99)	80.5 (73–105)	1.00
	FEV1	86 (63–102)	80 (64–100)	0.60
	FEV1/FVC	1 (0.9–1.1)	1 (0.8–1.2)	0.32
	DLCO	80 (74–99)	74 (50–87)	0.06

DLCO, diffusion capacity of the lung for carbon monoxide; FEV1, forced expiratory volume in one second; FVC, forced vital capacity. Data are presented as median (range), *p*-value is calculated using Wilcoxon rank sum test. * statistically significant.

**Table 4:** Comparison of eGFR (mL/min/1.73 m^2^) at the completion of chemotherapy and 1 year post-chemotherapy.

	Reduced Dose Bleomycin (*n* = 38)	Standard Dose Bleomycin (*n* = 7)	*p*-Value
At the completion of chemotherapy	126.5 (60–198)	119.0 (57–138)	0.287
One year post-chemotherapy	123.0 (71–169)	108.0 (96–134)	0.102
	**Cycles of chemotherapy ≤ 4** **(*n* = 19)**	**Cycles of chemotherapy > 4** **(*n* = 26)**	***p*-value**
At the completion of chemotherapy	132 (57–198)	119.5 (60–190)	0.171
One year post-chemotherapy	126 (98–169)	120 (71–153)	0.377

eGFR: estimated glomerular filtration rate; data are presented as median (range); *p*-value is calculated using Wilcoxon rank sum test.

## Data Availability

The data presented in this study are available on request from the corresponding author. The data are not publicly available due to restrictions (e.g., privacy or ethical).
